# Impact of Central Nervous System International Prognostic Index on the Treatment of Diffuse Large B Cell Lymphoma

**DOI:** 10.7759/cureus.16802

**Published:** 2021-08-01

**Authors:** Mohammad Ma'koseh, Faris Tamimi, Alaa Abufara, Lana Abusalem, Osama Salama, Yacob Saleh, Rnad Khader, Baha A Faiyoumi, Mohammad Al-Rwashdeh, Khaled Halahleh

**Affiliations:** 1 Medical Oncology, King Hussein Cancer Center, Amman, JOR

**Keywords:** diffuse large b cell lymphoma (dlbcl), central nervous system relapse, central nervous system international prognostic index, intrathecal chemotherapy, high dose methotrexate

## Abstract

Background

The central nervous system international prognostic index (CNS-IPI) is being used widely for the identification of patients with diffuse large B cell lymphoma (DLBCL) with a high risk of central nervous system (CNS) relapse. The aim of our study is to confirm the value of the CNS-IPI in predicting CNS relapse in our young study population and to evaluate its impact on the selection of patients for CNS prophylaxis.

Methods

We retrospectively reviewed patients diagnosed with DLBCL who were treated with rituximab, cyclophosphamide, doxorubicin, vincristine, and prednisone (RCHOP) regimen from January 2010 till December 2018. Correlation between CNS-IPI and cumulative incidence of CNS relapse and time to CNS relapse was examined through Kaplan-Meier plots. Median time to CNS relapse and median overall survival after CNS relapse were also estimated using the Kaplan-Meier plots.

Results

A total of 354 patients were included. The median age was 46 years. Overall, 5% of the patients developed CNS relapse. Median survival after CNS relapse was seven months. Two-year CNS relapse rates according to CNS-IPI were 0.7%, 5.1%, and 26% for low, intermediate, and high-risk, groups respectively.

On multivariate analysis, poor performance status (p=0.045), involvement of two or more extranodal sites (p= 0.021), involvement of bone marrow (p= 0.029), and renal or adrenal glands (p= 0.006) significantly correlated with CNS relapse. Considering the CNS-IPI and high-risk anatomical sites (breast, uterus, testis, and epidural space), 26% of our patients with DLBCL would have needed prophylaxis.

Conclusion

Although CNS-IPI helps in better selection of DLBCL patients for CNS prophylaxis, it can possibly increase the number of patients exposed to unnecessary prophylaxis. More investigational biomarkers are needed to better refining high-risk patients.

## Introduction

Diffuse large B cell lymphoma (DLBCL) is the most common lymphoma in adults, representing about one-third of newly diagnosed cases [1,2]. Using the standard combination chemo-immunotherapy rituximab, cyclophosphamide, doxorubicin, vincristine, and prednisone (RCHOP) regimen, 70% of cases are expected to be cured, while 30% will have the refractory or relapsed disease [3,4]. Although relapse in the central nervous system (CNS) is relatively rare, occurring in about 2-4% [5,6], it is a devastating event with a median survival of usually less than six months [7,8].

Many studies have attempted to identify risk factors for CNS relapse, with inconsistent results due to the heterogeneity in the patient population, limited sample size, and the fact that many were performed in the pre-rituximab era [9-11]. Accordingly, patients were variably selected for administration of CNS prophylaxis, mostly based on anatomical location (breast, testis, bone, cranial sinuses, and epidural space) and disease stage [12].

In 2016, Schmitz et al. developed the CNS-IPI using data of 2164 patients treated on prospective German High-Grade Non-Hodgkin Lymphoma Study Group (DSHNHL) studies and was validated in 1597 patients treated with RCHOP in British Columbia Cancer Agency (BCCA) [13]. Since then, the CNS-IPI has been adopted to evaluate the risk of CNS relapse by several national and international guidelines [14,15]. 

An accurate selection of the patients who need administration of various treatments to prevent CNS relapse is crucial, given the associated toxicities and the demand it causes on hospital services. The impact of CNS-IPI in selecting patients for screening of CNS involvement and administration of CNS-directed prophylaxis is not well studied. Our study aimed to validate CNS-IPI (as a separate scoring) in our patient population and to evaluate the indications for intrathecal chemotherapy (ITC) prophylaxis before the adoption CNS-IPI to estimate the impact of using CNS-IPI on the treatment of DLBCL.

This article was previously presented as a meeting poster at the Society of Hematologic Oncology (SOHO) 2019. Additionally, the article was previously published in Research Square. Ma'koseh M, Ma’koseh M, Tamimi F, et al.: Impact of central nervous system international prognostic index on the treatment of diffuse large b cell lymphoma. October 7, 2020. (DOI: 10.21203/rs.3.rs-81458/v1)

## Materials and methods

We retrospectively analyzed medical records of adult patients diagnosed with DLBCL without evidence of CNS involvement at diagnosis and treated with RCHOP regimen at the Department of Medical Oncology, King Hussein Cancer Centre in Jordan from January 2010 till December 2018. 

The following variables were retrieved from patients charts and electronic medical records: age, gender, Eastern Cooperative Oncology Group (ECOG) performance status, lactate dehydrogenase (LDH), albumin, alkaline phosphatase (ALP), stage, extranodal sites involved, the use of ITC, indications for ITC, systemic and CNS relapse.

The selection of patients for administration of CNS prophylaxis was mostly based on the discretion of the treating physician as the previous guidelines didn't specify specific indications. ITC was the only treatment given to prevent CNS relapse. 

The staging was done according to the Lugano staging system depending on computed tomography (CT) scan and positron emission tomography (PET-CT) scan (in patients diagnosed after 2012) [16]. The bulky disease was defined as tumor bulk more than 10 centimeters. Breast, testis, uterus, and epidural space were considered high-risk anatomical sites [9]. The refractory disease was defined as radiological evidence of disease progression during or within three months after finishing the last cycle of chemotherapy or radiotherapy, whereas relapsed disease was defined as radiological evidence of disease progression beyond three months after completion of therapy.

Patients were followed every three months in the first two years, then every six to 12 months thereafter, with clinical examination as well as CT scans for a total of five years after which, they were followed with clinical examination on a yearly basis. Lumbar puncture or brain imaging (CT or MRI) were done only in patients who developed symptoms of CNS relapse.

CNS relapse was diagnosed based on either the radiological findings, cerebrospinal fluid cytology, or brain biopsy. The CNS-IPI was calculated using age, stage, ECOG performance status, LDH, number of extranodal sites, and renal and adrenal gland involvement, and patients were classified as low risk, intermediate, or high risk, as previously described [13].

Correlation between the CNS-IPI and the cumulative incidence and time to CNS relapse was done through Kaplan-Meier plots. The correlation between different clinical and laboratory variables with time to CNS relapse was assessed by univariate and multivariate analysis utilizing the backward stepwise Cox-regression model.

The median time from DLBCL diagnosis to CNS relapse, survival from the diagnosis of CNS relapse till the last follow-up or death, survival patients who developed CNS relapse, and patients who didn't develop CNS relapse was calculated from the time of diagnosis till last follow-up or death and was plotted by the Kaplan-Meier method and compared by the logrank test.

## Results

Patients' characteristics and treatment

A total of 354 patients were included, 193 (54.5%) were males, with a median age of 46 (range, 18-90) years. CNS-IPI was low in 148 (41.9%) patients, intermediate in 161 (45.5%), and high in 45 (12.7%) patients (Table 1). Whereas 30 patients (8.5%) were considered to have high-risk anatomical sites (15 paraspinal, eight breast, four testicles, three uterus). In these patients, CNS-IPI was low in eight (26.6%), intermediate in 20 (66.7%), and high in two (6.7%) patients.

**Table 1:** Patients' characteristics ECOG PS: Eastern Cooperative Oncology Group performance status, LDH: lactate dehydrogenase, CNS-IPI: central nervous system international prognostic index, ITC: intrathecal chemotherapy, CNS: central nervous system

Feature	Number (%)
Age >60	98 (27.7%)
Gender	
Male	193 (54.5%)
Female	161 (45.5%)
B symptoms	143 (41%)
ECOG PS	
0-1	316 (89.3%)
>1	38 (10.7%)
High LDH	207 (59.3%)
Albumin <3.5 g/dl	53 (15.5%)
High alkaline phosphatase	62 (18.3%)
Bulky disease	114 (32%)
Stage	
I-II	140 (39.5%)
III-IV	214 (60.5%)
Extranodal involvement	230 (65.3%)
Number of extranodal sites	
< 2	266 (75%)
≥2	88 (24.9%)
Selected high-risk anatomical sites	
Renal or adrenal gland involvement	26 (7.3%)
Epidural mass	15 (4.3%)
Bone marrow	32 (9%)
Breast	8 (2.2%)
Uterus	3 (0.8%)
Testis	4 (1.1%)
CNS-IPI	
0-1 (low)	148 (41.8%)
2-3 (intermediate)	161 (45.5%)
4-6 (high)	45 (12.7%)
Intrathecal chemotherapy (ITC)	
Yes	52 (14.6%)
No	302 (85.4%)
Indications for ITC	
One high-risk anatomical site	
Skull bones and nasal sinuses	17 (32.7%)
Tonsils	5 (9.6%)
Epidural mass and spine	7 (13.5%)
Testicles	3(5.8%)
Kidneys	2 (3.8%)
Bone marrow/bone	6 (11.5%)
Multiple extranodal sites (including high-risk anatomical sites)	12 (23.1%)
CNS relapse	
Yes	17 (4.8%)
No	337 (95.2%)

All patients were treated with RCHOP. The number of chemotherapy cycles was based on the initial disease stage and interim radiological response, with a median of six cycles (range, 3-8). 

Two hundred and eighty-eight patients (81.3%) achieved a complete response (CR), and the rest were considered to have refractory disease. After a median follow-up of 27.7 months, 39(11%) patients developed relapse and 249 (70.3%) patients remained disease-free.

ITC with each cycle of chemotherapy was given to prevent CNS relapse to 52 (14.6%) patients. A median of four doses (range, 2-8) was given. The characteristics of the patients and the indications for ITC are detailed in Table 1. Sixteen patients (53%) with high-risk anatomical sites were given ITC.

CNS relapse

At a median follow-up of 27.7 months, 17 patients (4.8%) were diagnosed with CNS relapse. The median age was 31 years. The median time to CNS relapse was 6.9 months (range, 2-20). The CNS -IPI was low, intermediate, and high in one (5.8%), six (35.3%), and 10 (58.9%) patients, respectively.

CNS was the only site of relapse in six patients (35.3%), while it occurred before, concurrently with or after systemic relapse the remaining 11 patients (64.7%). Detailed characteristics of patients with CNS relapse are in Table 2.

**Table 2:** CNS relapse patients CNS-IPI: central nervous system international prognostic index, ITC: intrathecal chemotherapy, CNS: central nervous system

Feature	Number (%)
Median age (range)	31 (18-56)
Gender	
Male	10 (58.8%)
Female	7 (41.2%)
CNS-IPI	
Low	1 (5.9%)
Intermediate	6 (35.3%)
High	10 (58.8%)
Two or more extranodal sites	13 (76.5%)
Renal or suprarenal gland involvement	8 (47%)
Bone marrow involvement	5 (29.4%)
ITC prophylaxis	
Yes	5 (29.4%)
No	12 (70.6%)
Sites	
Parenchymal	9 (53%)
Leptomeningeal	6 (35.3%)
Both	2 (11.7%)
Isolated CNS relapse	6 (35.3%)
CNS relapse in the context of refractory systemic disease	7 (41.2%)
Simultaneous CNS and systemic relapse	2 (11.7%)
CNS relapse before systemic relapse	2 (11.7%)

**Figure 1:**
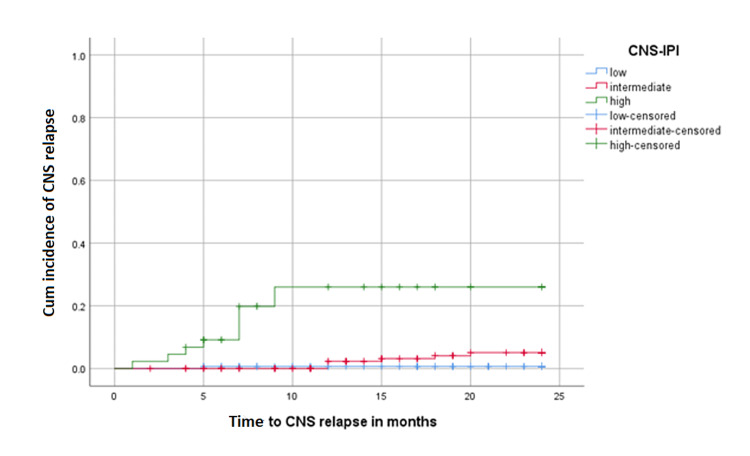
Cumulative incidence of CNS relapse according to CNS-IPI in all patients CNS: central nervous system, CNS-IPI: central nervous system international prognostic index

Among the whole group of patients, the two-year CNS relapse rates according to CNS-IPI were 0.7%, 5.1%, and 26% for low, intermediate, and high, respectively (p = <0001; Figure 1).

ECOG performance status of >1, involvement of two or more extranodal sites, high LDH, bulky disease, renal or adrenal involvement, and bone marrow involvement were associated with an increased risk of CNS relapse in univariate analysis, while ECOG performance status of >1, involvement of two or more extranodal sites, bone marrow, and renal or adrenal involvement were significantly associated with CNS relapse in multivariate analysis (Table 3).

**Table 3:** Univariate and multivariate Cox regression models for time to CNS relapse ECOG PS: Eastern Cooperative Oncology Group performance status, LDH: lactate dehydrogenase, ALP: alkaline phosphatase; CNS: central nervous system

Variable	Univariate analysis			Multivariate analysis		
	p-Value	Odds ratio	95.0% CI	p-Value	Odds ratio	95.0% CI
Male sex	0.656	1.25	0.47-3.27			
Age > 60	0.132	0.03	0-2.88			
B symptoms	0.204	1.90	0.71-5.1			
ECOG PS >1	0.001	5.71	2.1-15.5	0.045	3.05	1.02-9.1
High LDH	0.018	11.53	1.52-87.33	0.177	4.31	0.52-35.84
Albumin <3.5 g/dl	0.091	2.72	0.85-8.69			
High ALP	0.09	2.57	0.86-7.68			
Bulky disease	0.011	3.48	1.32-9.15	0.353	1.69	0.56-5.13
Stage III-IV	0.056	48.53	0.9-2608.05			
≥ 2 extranodal sites	< 0.001	11.42	3.72-35.07	0.021	5.61	1.29-24.32
Renal and/or adrenal involved	< 0.001	13.69	5.26-35.59	0.006	5.05	1.61-15.84
Bone marrow involved	0.005	4.39	1.54-12.45	0.029	3.45	1.14-10.49
Liver involved	0.683	1.36	0.31-5.95			

Among patients with high-risk anatomical sites (n=30), two patients (6.6%) developed CNS relapse; one with breast involvement who didn’t receive ITC and the other with epidural mass who received ITC.

ITC and CNS relapse

Among patients with CNS relapse, five patients (29.4%) were given ITC. When considering CNS-IPI risk groups, there was no significant difference in CNS-IPI risk categories in patients with CNS relapse given ITC (intermediate in one (20%) patient and high in four (80%) patients) or those with no ITC (low, intermediate and high in one (8.3%), five (41.7%0 and six (50%) patients respectively), p = 0.15. Among high-risk patients, there was no difference in CNS relapse between patients given ITC and the group who didn’t receive ITC (Table 4).

**Table 4:** ITC and CNS relapse among high-risk groups CNS-IPI: central nervous system international prognostic index, ITC: intrathecal chemotherapy

High-risk characteristic	Number	CNS relapse			No CNS relapse		p-Value
		ITC given		No ITC given	ITC given	No ITC given	
High CNS-IPI	44	4		6	4	30	0.064
Bone marrow involvement with low-intermediate risk CNS-IPI	25	0	2		6	17	1.000
Renal and adrenal gland involvement with low-intermediate risk CNS-IPI	2	0	0		1	1	NA
Breast, epidural space, testis, and uterine involvement with low-intermediate risk CNS-IPI	26	0	1		15	10	0.423

Survival

**Figure 2:**
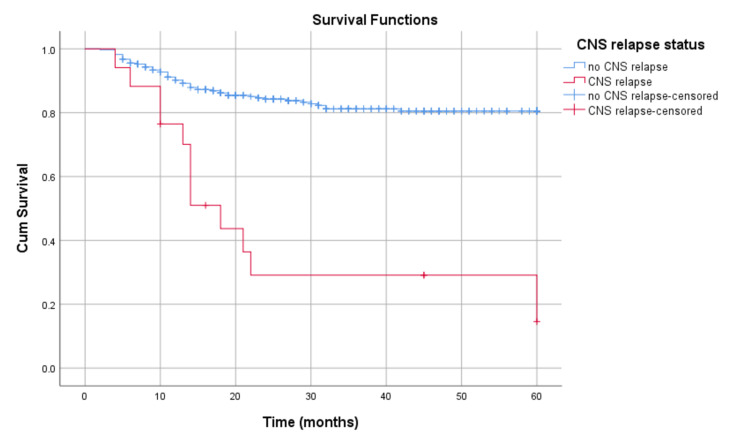
Survival of patients with CNS relapse vs no CNS relapse CNS: central nervous system

The median survival of patients with CNS relapse was 14 months (range, 6.9-21.1) while median survival after diagnosis of CNS relapse was seven months (range, 2-12). Five-year survival rate for patients with CNS relapse versus patients without CNS relapse is 14.6% versus 80.5%, respectively (p < 0.001; Figure 2). 

## Discussion

Analyzing the risk of CNS relapse in DLBCL is challenging as this is a rare event. The selection of patients for CNS prophylaxis is even more complex and depends on clinical and biological factors with varied indications in most of the published trials.

In five of the DHSNHL trials, prophylaxis was mandated for patients with bone marrow, testicular, or head and neck lymph nodes involvement [13] while in the United Kingdom National Cancer Research Institute (UK NCRI) trial, prophylaxis was given to patients with bone marrow, peripheral blood, nasal/paranasal sinuses, orbit, and testicular involvement [6].

In our study, 12.7% of patients had high-risk CNS-IPI, which is consistent with the data reported by Schmitz et al. [13]. Among this group, the two-year rate of CNS relapse in our study was relatively high (26 % compared to 12% in Schmitz et al.). Although we included a smaller number of patients, the risk of CNS relapse was variable even in the high-risk group; in our study, 15% and 32.5% of patients with CNS-IPI of five and six, respectively, developed CNS relapse [13].

We confirmed the value of CNS-IPI in predicting CNS relapse in a relatively younger age group as the median age of our patients is 15-20 years less than patients included in most of the studies published in this regard [6,11,13].

In our study, bone marrow involvement as well as multiple extranodal sites were associated with an increased risk of CNS relapse, which was observed in several trials [17,18] and in the BCAA confirmation cohort of Schmitz et al. trial, and explained by the exclusion of patients with >25% bone marrow involvement from DSHNHL trials [13].

In addition to high CNS-IPI, involvement of certain anatomical sites (breast, uterus, testis, and epidural space) may increase the risk of CNS relapse irrespective of the CNS-IPI [19-22]. Given the fact that the involvement of these sites is rare, they were underrepresented or even excluded from many prospective trials [9]. Guidelines vary in selecting these patients for CNS prophylaxis. For example, National Comprehensive Cancer Network (NCCN) guidelines recommend prophylaxis for patients with testicular, breast, and cutaneous DLBCL [14], while the Spanish lymphoma group recommends that patients with testicular, breast, kidneys, or adrenal glands and epidural space involvement should receive prophylaxis [15]. In our study, 30 (8.4%) patients had high-risk anatomical sites, among which CNS-IPI was high in two (6.6%) patients and CNS relapse occurred in two (6.6%) of the 30. Involvement of the tonsils and paranasal sinuses was associated with an increased risk of CNS relapse (6%) in the pre-rituximab era, but this risk decreased to 1.6% when rituximab was incorporated in the primary therapy [23].

Our data showed that 15% of the whole DLBCL patients were given ITC. However, if we included patients with bone marrow, renal, or adrenal glands involvement and low-intermediate risk CNS-IPI (27 patients; 7.3%) and patients with high-risk anatomical sites with low- intermediate risk CNS-IPI (26 patients; 7.3%) as candidates for CNS prophylaxis, a total of 97 (27.4%) patients with DLBCL would have been considered for CNS prophylaxis.

Despite the high correlation of CNS-IPI with the risk of CNS relapse, its positive predictive value in the original Schmitz et al. trial is low (12%), resulting in a significant proportion of patients that may unnecessarily receive prophylaxis [9,13].

The use of biomarkers may further help to identify high-risk patients. Two large studies evaluated the impact of the cell of origin (defined by gene expression profiling) on CNS relapse with conflicting results [24]. High-grade lymphomas with MYC and BCL 2 translocation (double hit) represent about 5% of all large B cell lymphomas with CNS involvement at diagnosis or relapse approaching 50% [25]. On the other hand, expression of MYC and BCL2 (double expressor) without translocation occurs in about 30% of DLBCL, the risk of CNS relapse appears to be increased in patients with activated B cell subtype and intermediate or high-risk CNS-IPI [26].

The best approach for the prevention of CNS relapse is still controversial because of the lack of well-randomized prospective trials, conflicting evidence, and potential toxicity. Although ITC is commonly used, evidence of efficacy is conflicting. Some studies showed that it is effective, but many failed to demonstrate a benefit, especially in high-risk patients [27]. The lack of efficacy of ITC may be due to the uneven distribution in the neuroaxis as well as the failure of significant penetration to the brain parenchyma as most relapses in the rituximab era are parenchymal rather than leptomeningeal [6]. Systemic high-dose methotrexate produces more equal concentrations in the subarachnoid space and has been shown to be effective in high-risk patients [28]. However, a recent study failed to show a benefit in high-risk patients (defined as high-risk CNS-IPI and double hit) [29], and there is still a debate regarding the optimal schedule and dose to be used [9].

Due to the small number of patients with high CNS-IPI given ITC in our study, we could not conclude on the efficacy of ITC. However, among patients with high CNS-IPI, the relapse rate appears to be high (four out of eight patients developed relapse). Our study has important shortcomings including the retrospective nature, not all patients included could be followed till the date of data collection, and the lack of data on the cell of origin and double hit or double expressor status.

## Conclusions

Inclusion of CNS-IPI in the evaluation of all DLBCL for deciding on CNS prophylaxis may help in better selection of patients with high risk for CNS relapse, but it can result in the exposure of many patients to unnecessary treatments. Further studies incorporating different biomarkers including the cell of origin and double hit or expressor subtypes are needed to help in the more proper selection of patients.
